# Contexto y Dinámicas de Atención Prenatal para Mujeres con Violencia de Pareja*

**DOI:** 10.15649/cuidarte.2118

**Published:** 2022-10-20

**Authors:** Cruz Deicy Jaramillo Bolívar, Gladys Eugenia Canaval Erazo

**Affiliations:** 1 Universidad Libre seccional Cali. Colombia. Email: cruzd.jaramillob@unilibre.edu.co Universidad Libre Universidad Libre Cali Colombia cruzd.jaramillob@unilibre.edu.co; 2 Universidad del Valle. Colombia. Email: gladys.canaval@correounivalle.edu.co Universidad del Valle Universidad del Valle Colombia gladys.canaval@correounivalle.edu.co

**Keywords:** Atención Prenatal, Violencia de Pareja, Embarazo, Salud de la Mujer, Personal de Salud, Prenatal Care, Intimate Partner Violence, Pregnancy, Women's Health, Health Personnel, Cuidado Pré-Natal, Violencia por Parceiro Íntimo, Gravidez, Saúde da Mulher, Pessoal de Saúde

## Abstract

**Introducción:**

Las mujeres tienen necesidades en salud que varían de acuerdo con el curso de vida y la violencia de pareja **í**ntima**.**

**Objetivo:**

identificar las características del contexto en el cual se brinda la atención prenatal a las mujeres identificadas con violencia de pareja.

**Materiales y Métodos:**

estudio cualitativo con el diseño de etnografía y observación participante focalizada. Realizado en las sesiones del curso de preparación para el parto y en salas de espera de cinco instituciones de salud en Cali, Colombia.

**Resultados:**

se identificaron tres dimensiones que sobre el contexto de atención: (a) El Ambiente donde se brinda la Atención, (b) Relaciones de poder y (c) Dinámicas de la atención. Los resultados muestran un contexto tradicional en la atención prenatal con predominio de un modelo biomédico.

**Discusión:**

Los hallazgos muestran la complejidad de la atención en las instituciones de salud y un contexto tradicional en la atención prenatal con predominio de un modelo biomédico.

**Conclusión:**

en el encuentro de las mujeres con el personal de salud no se identifica la violencia de pareja que sufren las mujeres, puesto que, la relación se ve medida por: desconocer necesidades, relaciones verticales, el poder y la comunicación no asertiva. Para la adopción de acciones de apoyo y acompañamiento, es necesario, reconocer la importancia de una atención en salud con enfoque de perspectiva de género, diferencial, e interseccional. Además, en favor de la autonomía y la dignidad de las mujeres se debe fortalecer la cultura del respeto y de empatía hacia las usuarias.

## Abstract (en)

**Introduction:**

Women have health needs that vary according to their life course and intimate partner violence.

**Objective:**

To identify the contextual characteristics in which prenatal care is provided to women with intimate partner violence.

**Materials and Methods:**

A qualitative ethnographic study based on participant-focused observation was conducted throughout childbirth preparation course sessions and waiting rooms in five healthcare institutions in Cali, Colombia. Results: The following three dimensions were identified in the care context: (a) envi ronment where care is provided, (b) power relations and (c) care dynamics. Findings suggest a traditional context in prenatal care with a predominance of a biomedical model.

**Results:**

three dimensions were identified regarding the care context: (a) The Environment where the Care is provided, (b) Power Relationships and (c) Dynamics of Care. The results show a traditional context in prenatal care with a predominance of a biomedical model.

**Discussion:**

The findings show the complexity of care in health institutions and a traditional context in prenatal care with a predominance of a biomedical model.

**Conclusions:**

During sessions held between women and healthcare personnel, intimate partner violence was not identified as the relationship was measured by a lack of knowledge of their needs, vertical relationships, power and non-assertive communication. For the adoption of support and accompaniment actions, it is necessary to recognize the importance of healthcare with a gender, differential and intersectional approach. In addition, in favor of women's autonomy and dignity, it is necessary to strengthen the culture of respect and empathy towards users.

## Resumo

**Introdujo:**

As mulheres tém necessidades de saúde que variam de acordo com sua trajetória de vida e violencia por parceiro **í**ntimo**.**

**Objetivo:**

identificar as características do contexto em que sao prestados cuidados pré-natais a mulheres identificadas com violencia do parceiro.

**Materiais e Métodos:**

estudo qualitativo usando etnografia e observado focalizada das participantes. Foi realizado nas sessóes do curso de preparado ao parto e nas salas de espera de cinco instituigóes de saúde em Cali, Colombia.

**Resultados:**

Foram identificadas tres dimensóes do contexto dos cuidados: (a) o ambiente onde os cuidados sao prestados, (b) as relagóes de poder e (c) a dinámica dos cuidados. Os resultados mostram um contexto tradicional nos cuidados pré-natais com predominio de um modelo biomédico.

**Discussao:**

Os achados mostram a complexidade do cuidado nas instituigóes de saúde e um contexto tradicional no pré-natal com predomináncia do modelo biomédico.

**Conclusao:**

no encontro entre as mulheres e o pessoal de saúde, a violencia dos parceiros sofrida pelas mulheres nao é identificada, uma vez que a relagao é medida por: ignoráncia das necessidades, relagóes verticais, poder e comunicagao nao assertiva. Para a adogao de agóes de apoio e acompanhamento, é necessário reconhecer a importáncia dos cuidados de saúde com uma perspectiva de genero, diferencial e intersetorial. Além disso, em prol da autonomia e dignidade das mulheres, é necessário reforgar uma cultura de respeito e empatia para com as pacientes.

## Introducción

Las mujeres tienen necesidades en salud que varían de acuerdo con el curso de vida, adicionalmente tienen otras necesidades que surgen de situaciones especiales, entre ellas, la Violencia de Pareja Íntima (VPI)^1^. La VPI sitúa a la mujer en condiciones de alta vulnerabilidad y en riesgo de adquirir padecimientos tales como alteraciones de la salud mental e infecciones de trasmisión sexual. Además, la VPI afecta en la mujer el ejercicio de sus derechos, sus potencialidades como ser humano, y su desempeño como pilar de la sociedad y agente de cambio^2^.

La VPI es una de las múltiples manifestaciones de la violencia de género, es considerada una violación de los derechos humanos y ha sido declarada un problema de salud pública^3^. Las consecuencias también son para los hijos, quienes al estar expuestos a eventos estresantes acarrean resultados funestos en su desarrollo^4^; algunas mujeres experimentan la VPI por primera vez durante la etapa de embarazo^5^ con consecuencias sobre el feto y el recién nacido.

La atención prenatal en Colombia es regida por el Ministerio de Salud y Protección Social; entre las normas establecidas está la Ruta de Atención Integral Materna y Perinatal, reglamentada por la Resolución 3280 de 2018^6^. Así, el sistema de salud tiene el compromiso de detectar, apoyar y hacer seguimiento a las mujeres identificadas con VIP; en esta medida el papel de los profesionales de la salud es fundamental en la atención a las mujeres gestantes y el de los profesionales de enfermería es crucial en el cuidado que dan a las mujeres en esta etapa de su vida^7^. La salud es un derecho y un componente del bienestar y de la calidad de vida de las personas; la atención en salud debe satisfacer las necesidades en salud; la búsqueda de rutina de la violencia contra las mujeres es mandatorio en todos los servicios de atención y en particular en la atención prenatal y postparto (Resolución 2626 de 2019)^8^.

Se ha estimado en diferentes regiones del mundo que entre 1.8% y 30% de las mujeres gestantes han experimentado violencia de pareja; en África y Latinoamérica las cifras son más altas que en Asia y Europa; en Colombia en 2000 y 2005 se reportaron datos de 8% y 10.7% respectivamente de mujeres gestantes que habían experimentado violencia de pareja^7^. Dentro de las manifestaciones de violencia de pareja, particularmente, la violencia física tiene efectos que influyen en el bienestar del producto del embarazo y la mujer misma, entre estos: el aborto, el bajo peso al nacer y el parto prematuro; en las mujeres, se presenta retraso en buscar atención prenatal, depresión antenatal o postparto, falta de ganancia de peso adecuado y menor tasa de lactancia^9^^-^^11^.

Con el propósito de aportar a la calidad de la atención de los servicios de salud se planteó la siguiente pregunta de investigación: ¿cómo es el contexto y la dinámica de los servicios de atención prenatal para mujeres gestantes que viven situaciones de violencia de pareja? Para dar respuesta a este interrogante, el objetivo de este estudio fue identificar las características del contexto donde se brinda la atención prenatal a las mujeres identificadas con violencia de pareja.

## Materiales y Métodos

Se empleó el diseño de etnografía focalizada, guiada por los lineamientos de la etnografía crítica^12^^)^ que orientó cómo realizar las observaciones de los lugares, los ambientes, las interacciones y las dinámicas de la atención. Se observaron los espacios donde se brinda la atención en salud como los consultorios y otros sitios en los cuales se mueven las mujeres usuarias de la atención, como son: los pasillos, los espacios de las instalaciones sanitarias, las salas de espera; también se observó el momento del control prenatal, la entrega de las citas de control o remisión, la toma de las muestras de laboratorio. También se observó a las personas usuarias de los servicios prenatales, a los proveedores de atención a la mujer gestante y las relaciones que se establecen entre el personal de salud y las mujeres.

La etnografía focalizada se caracteriza por visitas de campo cortas de gran intensidad cuyo foco es la recolección de datos sobre aspectos específicos como interacciones, acciones y situaciones particulares de instituciones u organizaciones altamente diferenciadas, de acuerdo con el propósito y objetivos del estudio; en donde la intensidad de las visitas aporta datos de diversa índole que son analizados de manera subsecuente ^13^.

El trabajo de campo se hizo con la revisión de documentos disponibles en el momento de la consulta prenatal y que portaban las mujeres durante las conversaciones con ellas en la sala de espera; mujeres que asistían a la consulta prenatal, de postparto, de crecimiento y desarrollo o de planificación familiar. Estas observaciones se hicieron en 5 instituciones de salud de carácter público y 1 institución privada, de manera intermitente en un período de 12 meses comprendido entre marzo de 2018 y abril 2019. De acuerdo con Guber^14^ observar es más que ver, es entender lo que se mira dentro del contexto en el que tiene lugar, identificando a las personas involucradas en producir y reproducir, crear y recrear, inventar y transmitir el sentido de aquello que experimentan. Se registraron notas de campo y se hicieron reflexiones acerca de lo observado en cada una y lo que podría ser observado en la siguiente visita. Se privilegió la perspectiva “emic” del punto de vista de las personas participantes.

El proyecto sombrilla, “Experiencia de la atención en salud en el control prenatal desde la perspectiva de mujeres en situación de violencia de pareja en Santiago de Cali, Colombia”; en el cual se enmarca este trabajo obtuvo la aprobación del Comité de Ética de la Universidad a la que pertenecen las investigadoras, aval consignado en el Acta No. 003-019R y de los Comités de Ética de las instituciones de Salud incluidas en el estudio.

El análisis se realizó siguiendo las directrices de Bradley, Curry y Devers^15^ aunque en investigación cualitativa no hay un modo correcto de analizar los datos y depende en gran proporción de la experiencia de quien realiza la investigación^16^.

Las notas de campo se leyeron tres veces para una inmersión profunda en los datos y encontrarle sentido a los mismos, se codificó manualmente las dimensiones emergentes, las dimensiones son los dominios conceptuales; se identificaron los temas de cada dimensión, los temas son conceptos unificados alrededor de los cuales se agrupan los datos y le dan un sentido general a los mismos a partir de la especificidad^15^, se seleccionaron los fragmentos o descripciones que servirían como citas para ilustrar lo hallado^17^. Se discutió entre las dos investigadoras las dimensiones y temas y se llegó a un acuerdo si existía diferencia; en la mayoría de los hallazgos hubo acuerdo desde el inicio.

## Hallazgos

Se identificaron tres dimensiones que dan cuenta de cómo es el *contexto de la atención,* como fenómeno en el que juegan papel fundamental el ambiente, las relaciones y las dinámicas de la atención en el Control Prenatal estas se denominaron así: (a) El Ambiente donde se brinda la Atención, (b) las Relaciones de poder y (c) las Dinámicas de la atención.

La Primera dimensión*, El Ambiente donde se brinda la Atención:* se refiere a los espacios y elementos alrededor de la planta física en el cual trabaja el personal de salud; como parte de esta dimensión se identificó el tema de ambiente físico, la incomodidad de la espera y comunicaciones en clave.

La segunda dimensión, *Relaciones entre profesionales y mujeres gestantes* **,** incluye las interacciones que se establecen entre los profesionales de salud y las mujeres y los aspectos que producen un efecto en la relación. Se identifican los temas, relaciones de poder, estrategias de coacción, invisibilidad de la experiencia y de la voz de las mujeres.

La Tercera dimensión, *Dinámica de la Atención Prenatal*, comprende las estrategias y acciones adoptadas por la institución y por quienes brindan la atención; incluye los temas denominados el modelo de atención biomédico, solo importa la productividad, seguir la rutina y normalización de la violencia.

### Ambiente donde se brinda la atención

*Ambiente Físico*: Se identificó que los espacios asignados para la atención varían dependiendo del tipo de servicio, público o privado. En los consultorios de carácter público observados, se encontró una serie de características como el poco espacio para la atención, los consultorios oscuros, con gran cantidad de elementos en desorden y poca limpieza, algunos de los equipos sin luminarias como lámparas de cuello de cisne, a otros les falta baterías o fallan debido a su mal estado. Estos espacios no aportan a la comodidad, no permiten establecer un ambiente de confianza e intimidad en la atención; adicional a lo anterior, las mujeres no tienen espacios amplios o cómodos para la espera que a veces se hace muy larga; en algunas salas de espera se escucha mucho ruido, por esto se agrupan en fila de manera estrecha en el pasillo contiguo al consultorio para escuchar el llamado y no perder el turno. En el consultorio de atención prenatal, se presentan interrupciones constantes adicional al ruido que proviene del pasillo. En otros sitios como la sala de espera que es para todo tipo de consulta, las sillas son insuficientes y el ruido “es molesto”, así lo manifestó una de las mujeres que esperaba a ser llamada.

Una profesional que atiende en el consultorio mencionó que escucha el ruido del pasillodonde se ubican las mujeres gestantes con sus acompañantes, comentó que todo se escucha dentro del consultorio, aún los comentarios que se refieren al personal de salud; se refirió así:

*“Yo ante esto, (se refiere al ruido en el pasillo) yo escucho cuando las mujeres afuera están hablando de mí y están diciendo que no hago, o que, si hago, que no las atiendo, o que si las atiendo: Las mujeres afuera creen que yo no las escucho y muchas lo que dicen no son cosas reales acerca de lo que está pasando, cuando entran les digo que las escuché, ellas se sonrojan, otras se sienten apenadas y algunas sencillamente no le dan importancia.”* (Profesional de salud)

*La Incomodidad de la Espera:* La sala de espera, es un espacio común donde se ubican las mujeres, algunas esperan paradas otras sentadas, este espacio común a todas las personas que asisten sin diferenciar a qué tipo de servicio consultan es pequeño, no es cómodo para sentar al número de personas que allí acceden; este espacio común a todos los usuarios se observa en las instituciones de las Empresas Sociales del Estado- ESES, allí están ubicadas las casillas marcadas con señales en las que se lee Caja, Citas, un poco más allá o al frente hay espacios con puertas y señales que indican el número del consultorio; se observan personas de diferentes características, hombres, y mujeres en su mayoría, son afrocolombianas y mestizas principalmente, varias personas adultas mayores, niños, niñas. Se ven largas filas para facturar (pagar por los servicios), en otros sitios se observa la fila de mujeres embarazadas quienes se ubican muy cerca al consultorio del control prenatal; no se encuentran suficientes sillas para sentarse, ni espacios dispuestos para la espera de manera cómoda, espera que en algunas oportunidades es de más de 45 minutos, lo que obliga a las mujeres y a sus acompañantes a esperar en el pasillo; allí las mujeres entablan conversaciones sobre diversos temas, entre esos asuntos sobre los que conversan, se escucha que hablan sobre la demora en la espera; hablan del personal que realiza las consultas y la manera como las atienden, otras conversaciones son sobre la crianza de los hijos, la planificación familiar y sobre las prácticas sexuales.

En una de las conversaciones con las mujeres en la sala de espera, una de ellas, comentó:

*“La espera el día de la consulta si es larga, un día tenía cita a las 11 am. Iban a ser como las 2 pm. y nada que me atendían, ese día jugaba Colombia, la médica se fue a ver el partido de Colombia (partido de fútbol) eso pasó una vez. Otro día vine a cita de control de Crecimiento y Desarrollo, esa cita era a la 1 pm y a las 2 pm de ver que no me atendieron yo hice el reclamo; la verdad es que tuve que irme porque no podía esperar más; la respuesta que me dieron es que teníamos que esperar”.* (Conversación con usuaria)

*Comunicaciones en Clave:* en las instituciones se observa un ambiente poco propicio para la atención a usuarios sin considerar que la gente que asiste en busca de servicios debe ser el centro de la actividad asistencial por encima de los trámites administrativos; las comunicaciones hacia los usuarios y el ambiente debe ser de respeto y de privacidad. Un aspecto observado se relaciona con la respuesta al llamado a la puerta según la clave que se use al tocar que le permite a quien está en el interior del consultorio, diferenciar si el sonido que produce es de las personas que esperan ser atendidas o de funcionarios de la institución.

Durante el proceso de la atención prenatal por la persona profesional de la salud a una mujer con cita previamente agendada, se presenció que durante la atención a la mujer gestante, tocaron la puerta del consultorio; la profesional de la salud quien atiende la consulta no respondió, parecía como si no hubiera escuchado el llamado a la puerta, ante la insistencia del llamado a la puerta, la observadora en ese momento (en el interior del consultorio ayudando en la consulta y a organizar el espacio) preguntó si abría la puerta, la profesional respondió, “*No, no es necesario, si fuera alguien importante yo lo sabría, entre la médica que atiende el control prenatal y el personal administrativo tenemos una clave para tocar*” (Nota de campo)

### Relaciones Entre las Mujeres Gestantes y los Profesionales

Las relaciones entre las mujeres gestantes y los profesionales son jerárquicas en las que se ejerce el poder del profesional sobre la mujer, y en ellas se impone autoridad, se coacciona y se invisibiliza la experiencia y la voz de las mujeres.

*Relaciones de Poder:* las profesionales de la salud ofrecen un trato poco cordial, regañan a las mujeres por diferentes razones, porque no se toman las muestras o no reclaman las ordenes o porque el resultado se alguna prueba de laboratorio salió positiva como sucedió con una persona a quien la prueba para VIH resultó positiva, y de manera poco prudente manifestó que debía inmediatamente hacerse otro examen de sangre y pasar a la consulta con el médico, esto lo mencionó en voz alta sin tener precaución de ser escuchada por las demás mujeres gestantes que se encontraban cerca; una de las personas que escuchó se mostró preocupada, se aproximó a mí con expresión de angustia en su rostro y me preguntó si a ella le pasaría lo mismo con su resultado, que ella no sabía de esa prueba porque era la primera vez que iba a control prenatal en esa institución. (Nota de campo)

*Medidas de Coacción:* las profesionales imponen condiciones para la continuidad de la atención a través de la entrega de la orden o el resultado de procedimientos como la ecografía al retener lo uno o lo otro y no entregarlos de manera oportuna. Lo entregan solo si la gestante asiste a la siguiente cita con otros profesionales diferentes al de medicina o si participan en el denominado curso de preparación para el parto, la maternidad y la lactancia; esta es una manera de presionar a que regresen a otras citas diferentes de la de medicina y si ellas cumplen le entregan el resultado; resultado es importante porque lo requiere llevar la mujer a la siguiente consulta con medicina.

Una profesional de la salud de una institución de salud dijo*:*

*“Mírela a ella.... nada que viene (se refiere a la mujer en embarazo), le he dado varias citaciones, y no cumple, entonces, que me quedó hacer como una opción para que la mujer regrese y asista al curso? quitarle la carpeta (se refiere a la historia clínica) y solo se la entrego hasta que ella asista al curso, y ahí si cuando llega al curso, le entrego la carpeta y también la orden de la ecografía; esta orden es para que realice los tramites de autorización para que le hagan la ecografía; con el resultado debe asistir a la siguiente cita de control que es con el medico; y continúa diciendo, porque eso es lo que les duele a las embarazadas, quieren saber si es niño o niña solo para empezar a buscarle nombre y para conseguir la ropa, ni siquiera es para que le digan que está bien, pues ellas “eso no lo entienden; esa estrategia si funciona” (retener la historia clínica y la orden de ecografía).* (Nota de campo)*.*

*Invisivilización de la Experiencia de las Mujeres:* En la consulta de atención prenatal de acuerdo con el protocolo establecido, el o la profesional pregunta, examina, escribe en el computador, no mira en muchas ocasiones la cara de la mujer sentada al frente, hace caso omiso sobre algún aspecto que ella desea ampliar, no le concede la palabra, no le contesta si está escribiendo en la historia clínica o llenando algún formato.

Una mujer gestante le expresó a la profesional sobre su condición de desplazamiento, la profesional continuó escribiendo sin ampliar sobre este asunto que pone a la mujer en condición de mayor vulnerabilidad; cuando la misma mujer quiso ampliar sobre lo que había pasado en su anterior embarazo cuyo resultado fue un bebe prematuro, la profesional no le contestó al respecto y ella, la gestante, tuvo que repetirle dos veces más y de manera sencilla y clara le expresó que era su intención dar a conocer su caso porque eso del niño prematuro, que era el menor que la acompañaba en la consulta, niño de aproximadamente 7 a 8 años, podría ser un dato de importancia para la historia clínica, la profesional le hizo alguna pregunta al respecto no dijo nada y continuó con la cabeza agachada escribiendo en la historia. (Nota de campo)

### Dinámicas de Atención

La atención prenatal se realiza por parte de profesionales de Medicina y de Enfermería según protocolos establecidos. En esta dimensión sobre las dinámicas de la atención, se observa el predominio del modelo biomédico, la productividad y la rutina, se normalizan los eventos de VPI que viven las mujeres, además la voz y la experiencia de las mujeres son invisibilizadas.

*Modelo Biomédico:* Las y los profesionales prestan la atención en el control prenatal bajo el modelo biomédico que ha sido el modelo predominante desde hace muchos años a pesar de los esfuerzos que hacen los entes territoriales, directivos de las instituciones y académicos por implementar otros modelos de atención. Se observó que la atención está centrada en la valoración de lo biológico, en la identificación de los síntomas físicos y los signos, en establecer un diagnóstico y la conducta a seguir para el tratamiento médico como terapia con medicamentos, remisión o educación sin tener en cuenta otras dimensiones de la persona.

*Seguir la Rutina:* Otro aspecto observado es la práctica rutinaria en el diligenciamiento de los formatos por parte de profesionales de la salud, quienes en muchas ocasiones omiten preguntas y se registran de manera automática algunos datos. Se observó que el registro en el sistema electrónico o en papel demanda tiempo; se diligencian varias pestañas y se escriben informes o llenan varios registros a la vez. El o la profesional de la salud se encarga de emitir ordenes, dar indicaciones para la autorización, la facturación y la formula o llenar el formato para solicitar los medicamentos.

Uno de los profesionales en Medicina mencionó:

*“Aquí hay que llenar la historia, yo tengo una hoja en Word con una proforma, de ahí copio y pego y solo cambio los datos correspondientes, eso es así para todas. Además, no hay tiempo, uno no pregunta sobre riesgo sicosocial, eso lo debe hacer psicología o enfermería; yo solo elaboro las ordenes de remisión a consulta de nutrición, odontología y psicología; y también entrego las órdenes para los exámenes de laboratorio de rutina para verificar que el embarazo siga sin complicaciones".* (Nota de campo - profesional de medicina en la consulta de atención prenatal)*.*

Las citas con otros profesionales diferentes a los de medicina y enfermería son consideradas de menor importancia, esto ocurre tanto para los prestadores como para las mujeres; las remisiones de rutina a los otros profesionales, diferentes de los mencionados, se hacen según lo que indica el protocolo; en algunos casos la remisión es opcional y no hay claridad del proceso a seguir. Una mujer gestante comenta sobre su caso:

*“Me remitieron a la psicóloga por rutina, recuerdo que registraron mis datos en la historia clínica pero nunca me dieron copia de la historia, si me dieron copia de la ecografía. No fui a la cita con la psicóloga porque cuando la solicité no había citas y yo no volví; me preguntaron porque no fui y dije, porque no había citas; tampoco fui a la nutricionista por la misma razón; es que cuando volví ya habían cerrado la carpeta, no había agenda*”. (Conversación con usuaria*)*

*Solo Importa la Productividad*: en reiteradas ocasiones, los profesionales de la salud detallaron, como una queja y con indignación, las dificultades que identifican en su práctica cotidiana, como también acerca de las rutinas para la atención y la necesidad de cumplir indicadores de atención, dada la productividad exigida en las instituciones. Estas situaciones producen un efecto en los tiempos, los cuales se acortan para la consulta y para los trámites administrativos, también se afecta el tiempo para la entrega de la información a las usuarias, aspecto que incide en la calidad de la atención. Una profesional que realiza los controles prenatales en una de las observaciones refirió:

“*Me angustia lo administrativo de aquí, meten pacientes cada 20 minutos y en ese tiempito uno no alcanza a explicar nada. Ahora también han cogido que si vienen los pacientes sin cita los ponen a esperar y si a uno le queda un tiempito los hacen facturar para atenderlos, eso así no sirve, uno no puede ni leer la historia clínica".* (Profesional de medicina del servicio prenatal)

Como consecuencia de lo anterior los profesionales ajustan el tiempo de duración de la consulta, responden por la valoración biomédica de la usuaria, en medio de una rutina que permita optimizar el tiempo requerido y dar salida a la atención de las mujeres; en ese sentido, en las consultas no se les pregunta a las mujeres, de manera sistemática, por los riesgos psicosociales y no hay un ambiente de confianza para expresar necesidades de cuidado ni sobre la situación de VPI que viven las mujeres, no hay abordaje de aspectos relacionados con la salud mental, una profesional expresó: *“El control tiene su secuencia, y solo si ves cosas muy graves diferentes a alteraciones del embarazo normal, realizas una remisión; el tiempo no da para más, cuando miras las citas en el computador tienes programadas muchas citas”.* (Profesional de medicina del servicio prenatal).

*Normalización de la Violencia:* las mujeres se expresan acerca de la violencia psicológica y emocional como algo que es normal que ocurra en las relaciones de pareja. Los profesionales de la salud no identifican la VPI, es algo sobre lo que no se pregunta, como si también les pareciera que es normal. En una observación en uno de los sitios donde se realizaba el control prenatal se encontró que algunas mujeres con sus hijos en control de crecimiento y desarrollo o para planificación familiar relataron lo siguiente:

*“Si, en el control prenatal me preguntaron por enfermedades en la familia y las mías;* Mencionó*: no recuerdo que me hayan preguntado por las relaciones con mi pareja, ni sobre situaciones de riesgo psicosocial.* (Notas de campo y conversaciones con las mujeres de la sala de Espera)

Otra mujer refirió que sí le preguntaron sobre las relaciones de pareja, que ella dijo que eran discusiones que se presentaban con el esposo de vez en cuando, al insistirle sobre cuantas eran las veces de las discusiones y cómo eran en las mismas*,* respondió*:*

*“no siempre se presentaron, [,..]era lo "normal". “Es lo normal.* Él *no me tira, son discusiones de vez en cuando [...] en el control no me recomendaron cuál era el camino para seguir si las discusiones con él (esposo) empeoraban, ni a donde acudir [...] aunque escuché que a otras personas con problemas las llaman al celular para darles cita [...] si me explicaron sobre la lactancia"* (Notas de campo y conversaciones con las mujeres de la sala de Espera)

## Discusión

Los hallazgos muestran la complejidad de la atención en las instituciones de salud y un contexto tradicional en la atención prenatal con predominio de un modelo biomédico en el cual prevalecen ambientes inadecuados para la atención, con relaciones de poder y dinámicas de atención con medidas de coacción por parte de los profesionales de la salud sobre las mujeres.

El control prenatal es la puerta de entrada de muchas mujeres a los servicios de salud así que este espacio se constituye en una ventana de oportunidad de atención para identificar a las mujeres con necesidades especiales, prescribir el tratamiento, dar orientación, apoyo y seguimiento^18^^)^ en concordancia con el Modelo de Atención Integral Territorial (MAITE) que articula el ente gubernamental nacional con el ente territorial, en Colombia^8^.

Los resultados de este estudio exponen la existencia de contextos y dinámicas de atención basadas en relaciones de poder que repercuten en la autonomía y la agencia de las mujeres; los ambientes generan interacciones fugaces, distantes, que no crean confianza, por el contrario, conducen al disciplinamiento de las mujeres.

En este tipo ambientes la primacía es la norma, la rutina y la atención centrada en detectar cualquier anormalidad o enfermedad asociada al embarazo; las mujeres gestantes no compartirán sus situaciones de violencia de pareja si en los momentos de la atención los profesionales no disponen de tiempo, no se establece una relación de confianza y empática, y no se cuenta con espacios en los cuales se respete la privacidad. Es mandatorio identificar las barreras del contexto y las propias de los profesionales que impiden que las mujeres gestantes manifiesten sus emociones, sentimientos, necesidades y que pregunten sobre qué hacer ante su situación de violencia de pareja. En consecuencia, el trabajo de profesionales de enfermería es fundamental para el establecimiento de momentos de cuidado; momentos que hagan una diferencia e innoven en la atención en salud con la participación de las mujeres gestantes, sus familias y la comunidad.^19^^,^^20^

El cuidado que responde a las necesidades de las personas tiene que ser brindado por profesionales de la salud competentes, con formación en un amplio número de teorías, entre ellas las del cuidado, las de vulnerabilidad social, las de promoción de la salud y las de la competencia cultural^21^. También, se requiere que el profesional desarrolle un compromiso afectivo con el otro, en otras palabras, una actitud que deriva en acciones a través de las cuales se expresa la disposición e interés por el otro, de acuerdo con Boff se hace indispensable la necesidad de cuidar de la espiritualidad humana^22^. En muchas ocasiones para las personas que viven en situación de VPI el único contacto al cual acudir es el proveedor de servicios de salud; por ello es importante que los profesionales y técnicos, de los servicios de salud pregunten activamente por la existencia de VPI, escuchen las experiencias de las mujeres y provean cuidados^23^.

En el campo de atención psico-social y del cuidado en salud se ha encontrado que el preguntar y captar las emociones; encontrar pistas que les permita sospechar acerca de la situación particular de la usuaria; además, el no centrarse solo en los síntomas y disponer de tiempo, son componentes de la atención en modelos diferentes al modelo biomédico^24^. Por el contrario, en el encuentro de las mujeres con los profesionales se pasan por alto las necesidades propias de las mujeres, se establecen relaciones verticales, mediadas por el poder y la comunicación que no reconoce la situación de VPI y que al no identificarla implica la falta de adopción de acciones de apoyo y acompañamiento, de remisión, y de atención especializada. Surge la pregunta sobre si conocen los profesionales y en particular los de enfermería el cómo actuar en relación con todas las dimensiones de cuidado humano^25^. Algunas acciones buscan disciplinar a las mujeres de acuerdo con las necesidades de los servicios o de recabar los indicadores esperados; el disciplinar a las mujeres es una práctica de siglos pasados, que aún prevalecen. como lo reportan diversos autores en diferentes áreas^26^.

Facilitar la participación de las mujeres gestantes y sus familias, en su proceso de atención prenatal puede ayudar a reducir el desbalance de poder, a crear conocimiento, a tomar decisiones conjuntas con los profesionales de la salud y a definir acciones que propendan por resultados socialmente justos y equitativos^27^^-^^30^.

La teoría de mediano rango sobre transiciones y la terapéutica del cuidado indica que las mujeres presentan expectativas, en el tiempo del embarazo su identidad fluida se torna diferente, se requiere ganar maestría, ampliar sus habilidades de crianza, y obtener un resultado de ese tránsito que genere salud y bienestar. Los profesionales de la salud tienen un papel fundamental en los procesos de cambio de la atención en salud a mujeres gestantes las cuales viven una etapa de transición, que requiere de ajustes y de acciones que las ayude y las conduzca a una transición saludable^26^^,^^31^ y en particular a las mujeres gestantes en situaciones especiales como ocurre con mujeres en situación de violencia de pareja.

La comunicación es elemento importante para el cumplimiento del rol en los profesionales^32^, independientemente del campo de acción en el cual se desempeñen sea enfermería, medicina, psicología u otras. La comunicación debe ser efectiva, clara, que oriente y apoye; en este estudio *se* encontró que en la relación profesional - mujer gestante no se establece una comunicación efectiva, aspecto a considerar para propender por un cambio de paradigma en los modelos de atención.

Se encontró en el presente estudio que aún continúa privilegiándose el modelo biomédico y poco o nada se tiene de en un modelo centrado en el paciente, en la persona y en este caso con la mujer gestante; sin embargo, se encuentran variaciones según los contextos culturales y las condiciones laborales, de allí la importancia de comprender la realidad que se vive en los servicios de salud. La comunicación es esencial en el momento de brindar un cuidado holístico a las mujeres gestantes como ha sido pregonado en ámbitos particulares de cuidado ante personas con situaciones de salud o de enfermedad. El manejo de la comunicación por parte de los profesionales requiere de estrategias de intervención desde la cultura organizacional y la adopción de un modelo de cuidado de acuerdo con Thorne *et al*.^33^ e n el cual el respetar la dignidad de la persona sea un derecho fundamental^34^.

Por otra parte, la sobrecarga de trabajo y el aumento de las tareas administrativas de los profesionales a expensas del tiempo para la atención, afecta las relaciones de comunicación con los pacientes, esto se encontró en un estudio con pacientes con cáncer que el observar a las profesionales con múltiples tareas y poco tiempo para los pacientes les indicaba una sobrecarga de trabajo y produjo que los usuarios se abstuvieran de hablar de sus emociones y sentimientos^32^. Las mujeres necesitan contar con profesionales que dispongan de tiempo y brinden un ambiente de confianza y seguridad para que ellas puedan expresar sus emociones y situaciones particulares como la violencia de pareja íntima sin temor a ser cuestionadas o revictimizadas.

Identificar a las mujeres con situaciones de VPI y cuestionar por qué se continúa normalizando los eventos de violencia de pareja es prioritario. Los profesionales de la salud y en particular enfermería deben ser capacitados para brindar un cuidado compasivo que facilite la empatía, la confianza entre el proveedor de servicios y las mujeres gestantes, que les ayude a ellas a reportar su situación y a la búsqueda de apoyo social^35^. El apoyo social a las mujeres gestantes en situación de VPI es parte del ejercicio profesional en salud en tanto las instituciones de salud tienen que actuar como protectores de la salud, activar la ruta de atención de violencias y brindar un cuidado que propenda por la preservación o restauración de los derechos humanos y la dignidad de las personas, sumado a la práctica de detección de riesgo de enfermedad, el diagnóstico precoz y tratamiento oportuno y adecuado.

El apoyo a las mujeres para desarrollar competencias para la crianza es parte de lo que la atención en salud debería ocuparse en concurrencia con otros sectores como bienestar social y educación. La VPI no solo tiene impacto en la mujer que la padece, sino que se ha documentado que mujeres en situación de VPI tienen dificultades con la crianza de los menores^36^; así, las consecuencias psicosociales de la VPI son nefastas en los hijos. Investigaciones en el tema reportan que los hijos pueden presentar síntomas de estrés postraumático; baja competencia social; problemas emocionales y conductuales; un mayor riesgo de presentar conductas externalizantes (hostilidad y agresión) o un mayor riesgo de conductas internalizantes (autoculpabilización y vergüenza)^37^^-^^40^.

Como *reflexión final*, se debe mencionar que esta experiencia investigativa aporta a la comprensión de la atención en salud, dando vida a la voz de los actores. Sumado a ello, se identificó que la atención en salud a las mujeres no es centrada en ellas mismas, ni en sus necesidades. Urge mirar como un reto y una oportunidad para cambiar el modelo de asistencia biomédico, el cual implica relaciones jerárquicas de poder sobre las mujeres y con expresiones de violencia hacia ellas que obedece a un problema sociológico de los servicios de salud y de sus actores que a su vez se han formado en un modelo educativo de jerarquía^41^.

El cambio debe efectuarse hacía un modelo de cuidado, el cual se centre en las necesidades de las personas, que sea un modelo integrado y continuo como el planteado por Forbes y Neuhfeld^42^^,^^43^ que incluya el trabajo conjunto y coordinado del equipo interdisciplinario^19^; la integración de los servicios médicos, de enfermería, de otros profesionales de la salud en un mismo sitio; con facilidad en acceso a ayudas diagnósticas, educación en salud y al apoyo social, entre otros.

El sistema de atención en salud le da menos énfasis al cuidado de los seres humanos, Watson^19^^)^ llama la atención porque éste sea restaurado si queremos que el sistema de salud sobreviva de manera ética y responsable y en particular si la enfermería cuyo fundamento de su conocimiento y de su práctica es el cuidado, perdure como una profesión única que cumple con el mandato oficial de dar cuidado. Se requiere una atención en salud que incluya el cuidado transpersonal y culturalmente competente como lo han señalado Watson^20^, Meleis^44^ y Leininger^45^ en el que se avance hacía un ambiente de cuidado en el cual se desarrolle un proceso de relaciones con las mujeres gestantes que sea respetuoso de sus valores, de la diversidad cultural y étnica entre otros.

En suma, un modelo en salud que permita la expresión de sentimientos y necesidades, que trascienda la valoración de síntomas físicos; que potencie la paz, la totalidad y la dignidad; y privilegie la participación de las mujeres, sus familias y la comunidad; la educación en salud, el reconocimiento de los recursos de la comunidad y el acceder a ellos. Que además propenda por el desarrollo de la autonomía y el ejercicio de la agencia de las mujeres; que elimine las estructuras de poder y las relaciones de autoritarismo en donde las mujeres y sus familias tengan una voz^46^^,^^47^.

## Conclusión

Los resultados muestran un contexto tradicional en la atención prenatal con predominio de un modelo biomédico en el cual prevalecen ambientes inadecuados para la atención, se observaron relaciones de poder y dinámicas de atención con medidas de coacción sobre las mujeres por parte de los profesionales de la salud. También, se mantiene la rutina en la atención con centralidad en la productividad y no en las mujeres usuarias del servicio, lo que va en detrimento de la calidad de la atención.

Se recomienda que desde la formación de los profesionales de salud se reconozca la importancia de realizar una atención en salud que sea diferencial, con perspectiva de género, bajo la mirada de la interseccionalidad para tener en cuenta condiciones de vulnerabilidad como etnia, edad, condición de desplazamiento, situación de VPI, posición social, diversidad funcional, entre otros. Asimismo, que se fortalezca la cultura del respeto tanto en el proceso de formación de los profesionales^48^ como en las instituciones de salud y se brinde atención con empatía hacia las usuarias en favor de la autonomía y la dignidad de las mujeres^49^.
